# Association between community medicine rotation duration and general medicine in-training examination scores among second-year resident physicians in Japan: a nationwide cross-sectional study

**DOI:** 10.1186/s12909-026-09897-y

**Published:** 2026-07-07

**Authors:** Kiyoshi Shikino, Yuji Nishizaki, Miwa Sekine, Nobuyuki Araki, Naoto Ozaki, Sho Fukui, Yu Yamamoto, Taro Shimizu, Hiroyuki Kobayashi, Yasuharu Tokuda

**Affiliations:** 1 Department of Community-Oriented Medical Education, Chiba University Graduate School of Medicine, 1-8-1 Inohana, Chuo-ku, Chiba City, Chiba Prefecture Japan; 2 Department of General Medicine, Chiba University Hospital, Chiba, Japan; 3 Division of Medical Education, Juntendo University School of Medicine, 2-1-1, Hongo, Bunkyo-ku, Tokyo Japan; 4 General Medicine, Kyorin University School of Medicine, Tokyo, Japan; 5 Division of General Medicine, Center for Community Medicine, Jichi Medical University, Tochigi, Japan; 6 Department of Diagnostic and Generalist Medicine, Dokkyo Medical University Hospital, Tochigi, Japan; 7 University of Tsukuba, Tsukuba, Ibaraki Japan; 8 Department of Internal Medicine, Mito Kyodo General Hospital, Tsukuba, Ibaraki Japan; 9 Muribushi Okinawa Center for Teaching Hospitals, Okinawa, Japan; 10 Tokyo Foundation for Policy Research, Tokyo, Japan

**Keywords:** community medicine, postgraduate medical education, residency training, General Medicine In-Training Examination, general medicine, Japan

## Abstract

**Background:**

Although community medicine is a required component of postgraduate clinical training, its actual duration and relation to knowledge-based performance remain unclear. This study examined the national distribution of community medicine rotation duration and its association with General Medicine In-Training Examination (GM-ITE) performance.

**Methods:**

We conducted a nationwide multicenter cross-sectional study using GM-ITE 2024 data from second-year resident physicians. Outcomes were compared between those with 1 month and those with 2–3 months of community medicine training. The primary outcome was the GM-ITE total score rate (%), and the prespecified secondary outcome was the correct rate (%) in the general medicine category. Other domain- and specialty-specific correct rates were examined descriptively. Multivariable linear regression was used to estimate the associations between community medicine rotation duration and total score rate.

**Results:**

The study included 2,834 second-year residents. Community medicine rotation duration was 1 month for 2,243 residents (79.1%), 2 months for 435 (15.3%), 3 months for 67 (2.4%), 4 months or more for 21 (0.7%), and missing for 68 (2.4%). Total score rate was similar between the one-month and 2–3-month groups (58.36±10.11 vs 58.50±10.23; p=0.785; Cohen’s d=0.01). The general medicine correct rate was nominally higher in the 2–3-month group than in the one-month group (54.43±18.31 vs 52.49±17.37; *p*=0.031; Cohen’s d=0.11), but this difference did not remain statistically significant after false discovery rate adjustment (q=0.122). In the multivariable analysis restricted to residents with 1–3 months of community medicine rotation and complete covariate data, a two-month rotation was not significantly associated with total score rate (adjusted β: 0.44; 95% CI: −0.54 to 1.42; p=0.382), whereas a small adjusted association was observed for a three-month rotation (adjusted β: 2.33; 95% CI: 0.29 to 4.37; *p*=0.025).

**Conclusions:**

One month was the predominant duration of community medicine training in Japan. A small, adjusted association with the total GM-ITE score rate was observed for a three-month rotation but not for a two-month rotation, and it should be interpreted with caution. The general medicine finding was small, exploratory, and not statistically significant after adjustment for false discovery rate.

**Supplementary Information:**

The online version contains supplementary material available at https://doi.org/10.1186/s12909-026-09897-y.

## Background

Internationally, community-based and rural training has been used to expose trainees to underserved settings, strengthen preparedness for community-oriented practice, and address geographic maldistribution of physicians [1–3]. Since mandatory postgraduate clinical training was introduced in 2004, Japan has emphasized the acquisition of broad clinical competence during the two-year initial residency period, regardless of future specialty choice [4–7]. Within this framework, community medicine training is intended to expose resident physicians to patient care across diverse community settings and to help them understand continuity of care, home care, discharge support, and collaboration with multiple professionals and community resources [8–10]. Community-based medical education in Japan has also been discussed in relation to community comprehensive care education and interprofessional education, and prior studies have shown that community and rural placements can help trainees understand community health, family-oriented care, and the social context of patients [11–14]. These learning opportunities are closely aligned with Japan’s community-based integrated care system, which emphasizes coordination among medical care, long-term care, preventive care, housing, and livelihood support in aging communities [15, 16]. In Japan’s super-aged society, community medicine training is particularly relevant because physicians are expected to understand not only disease management but also long-term care, home care, preventive care, and coordination with community resources. Because community medicine rotations often involve multiprofessional collaboration, they also overlap conceptually with interprofessional education and collaborative practice [17, 18]. Because the initial residency period is limited, extending community medicine rotations may reduce the time available for other required training experiences. Determining an appropriate duration for community medicine training is therefore an important issue in postgraduate medical education.

The General Medicine In-Training Examination (GM-ITE) is a nationwide standardized examination used to assess residents’ clinical knowledge and reasoning in Japan [19, 20]. It covers multiple competency domains, such as general medicine, clinical reasoning, physical examination, clinical skills, and disease-specific knowledge, with some items delivered in video or audio format [19, 20]. Previous GM-ITE studies have shown that examination performance is associated with the educational environment, hospital volume, hospital type, duty hours, general medicine rotation training, internal medicine rotation length, and outpatient training [21–27]. These findings suggest that associations between training duration and examination performance may differ according to the type of educational experience and the outcome being measured. However, no nationwide study has specifically examined whether the duration of community medicine rotation is associated with resident physicians’ GM-ITE performance. In particular, it remains unclear whether longer community medicine rotations are associated with higher overall GM-ITE scores or with better performance in the general medicine domain, which may be more closely aligned conceptually with community medicine training. This study, therefore, aimed to describe the national distribution of community medicine rotation duration and to examine its association with GM-ITE scores among second-year resident physicians in Japan.

## Methods

### Study design and setting

We conducted a nationwide, multicenter, cross-sectional study using data from the GM-ITE 2024. The GM-ITE is administered voluntarily to resident physicians at participating clinical training hospitals throughout Japan. For PGY-2 residents, the GM-ITE is taken near the end of the two-year initial postgraduate clinical training program, allowing assessment of examination performance at the completion stage of initial residency. Examinees complete a background questionnaire on the day of the examination. The background questionnaire was not developed specifically for the present study; it is the standard questionnaire administered by the Japan Institute for Advancement of Medical Education Program (JAMEP) as part of the GM-ITE and has been used in previous GM-ITE studies [19–27]. An English-language version of the questionnaire items used in the present analysis is provided as Supplementary file 1. This study proceeded in accordance with the Strengthening the Reporting of Observational Studies in Epidemiology (STROBE) statement for cross-sectional studies [28].

### Participants

The study population comprised postgraduate year-two (PGY-2) resident physicians who participated in the GM-ITE 2024 and provided consent for their data to be used for research purposes. We focused on PGY-2 resident physicians because community medicine training is typically completed within the two-year initial residency program and because end-of-training performance was our primary interest. A total of 2,834 resident physicians were available for the overall descriptive analysis. For the principal comparison between 1 month and 2–3 months of community medicine training, resident physicians who did not respond to the rotation duration item were excluded due to missing data. In addition, those reporting 4 months or more of community medicine training were excluded from the principal comparative analyses because these responses were rare and may have reflected atypical program structures or heterogeneous interpretations of the questionnaire item. After these exclusions, 2,745 resident physicians remained for the principal comparative analysis. For multivariable regression, we performed a complete-case analysis, resulting in 2,689 resident physicians.

### Exposure: community medicine rotation duration

The primary exposure was the self-reported duration of community medicine rotation in the background questionnaire. Four weeks were treated as one month. We first described the full national distribution of community medicine rotation duration. Since only a small number of residents reported more than three months of community medicine training, the principal comparative analyses were restricted to residents with 1–3 months of training. For descriptive comparisons, we classified residents as having completed one month versus 2–3 months of community medicine training. In multivariable regression, rotation duration was modeled as a categorical variable, with one month as the reference category and separate indicators for two and three months.

The comparison between one month and 2–3 months was selected because one month corresponds to the minimum required duration of four weeks in the Japanese postgraduate clinical training guideline, whereas two to three months corresponds to longer 8- to 12-week rotations that some programs may implement, and is consistent with the guideline statement that eight weeks or more is desirable [29].

### Outcomes

The primary outcome was the GM-ITE total score rate (%). The total score rate was calculated as the percentage of the maximum possible GM-ITE total score. The prespecified secondary outcome was the correct rate (%) in the general medicine category, because this category was considered conceptually aligned with the educational aims of community medicine training. We also descriptively examined other domain-specific correct rates for clinical reasoning, physical examination/procedures, and specific disease, as well as specialty-specific correct rates for internal medicine, surgery, pediatrics, obstetrics and gynecology, psychiatry, and emergency medicine.

We selected the total score rate as the primary outcome because it is a standardized nationwide measure of knowledge-based clinical performance among resident physicians in Japan and enables comparison with previous GM-ITE studies. However, GM-ITE does not directly assess all competencies targeted by community medicine training, such as interprofessional collaboration, continuity of care, communication skills, home care, discharge planning, and community-oriented practice.

### Covariates

Covariates were selected a priori based on previous GM-ITE studies examining resident physicians and training-environment factors [19–27]. The final multivariable model included age, gender, weekly work hours, on-call frequency, self-study time, hospital type, and regional quota status, in addition to community medicine rotation duration.

### Statistical analysis

We summarized resident characteristics, community medicine rotation duration, and GM-ITE outcomes using descriptive statistics. Categorical variables are presented as numbers and percentages, and continuous variables are presented as means and standard deviations. The overall distribution of community medicine rotation duration was described among all eligible PGY-2 resident physicians.

For the principal comparative analyses, residents were categorized into two groups according to community medicine rotation duration: one month and 2–3 months. Residents with missing rotation duration data and those reporting four months or more of community medicine training were excluded from these principal comparative analyses. Differences in the distribution of community medicine rotation duration by gender, hospital type, and regional quota status were examined using chi-square tests or Fisher’s exact tests, as appropriate.

GM-ITE total score rates and domain- and specialty-specific correct rates were compared between the one-month and 2–3-month groups using Welch’s t-tests. We also presented GM-ITE outcomes separately for residents with one-, two-, and three-month rotations, using Welch one-way analysis of variance for overall comparisons and Welch’s t-tests for pairwise comparisons with the one-month group as the reference. Domain-specific correct rates included general medicine, clinical reasoning, physical examination/procedures, and specific disease. Specialty-specific correct rates included internal medicine, surgery, pediatrics, obstetrics and gynecology, psychiatry, and emergency medicine. Correct-rate comparisons were interpreted as exploratory. Nominal p-values are reported, and Benjamini-Hochberg false discovery rate adjustment was additionally applied separately to the exploratory domain-specific and specialty-specific correct-rate comparisons as a sensitivity analysis. The GM-ITE total score rate was treated as the primary outcome and was not included in the FDR correction. For the FDR analyses, q < 0.05 was considered statistically significant. For GM-ITE outcomes, unadjusted mean differences, 95% confidence intervals, and Cohen’s d were calculated to describe the magnitude of between-group differences.

For the primary adjusted analysis, we constructed a multivariable linear regression model with the GM-ITE total score rate (%) as the dependent variable. This model was restricted to residents with 1–3 months of community medicine rotation and complete covariate data, consistent with the principal comparative analyses. Community medicine rotation duration was modeled as a categorical variable, with one month as the reference category and separate indicators for two and three months. The model adjusted for age, gender, weekly work hours, on-call frequency, self-study time, hospital type, and regional quota status. Adjusted regression coefficients (β), 95% confidence intervals (CIs), and p-values are reported. Robust standard errors were used to calculate 95% CIs and p-values. Complete-case analysis was used for the multivariable model. As sensitivity analyses, we repeated the multivariable regression models using hospital-level cluster-robust standard errors and after restricting the sample to non-university hospitals. We also compared residents included in the complete-case regression analysis with those excluded because of incomplete covariate data.

No formal sample size calculation was performed because this was a secondary analysis of all consenting PGY-2 resident physicians with available GM-ITE 2024 data. All tests were two-sided, and *p* < 0.05 was considered statistically significant for nominal analyses. All analyses were performed using R version 4.4.1.

### Ethical approval

This study was approved by the Ethics Review Committee of the Japan Institute for Advancement of Medical Education Program (JAMEP) (approval no. 25-3). Informed consent for research use was obtained from examinees at the time of taking the GM-ITE.

## Results

### Participants and the distribution of community medicine rotation duration

**Fig. 1:**
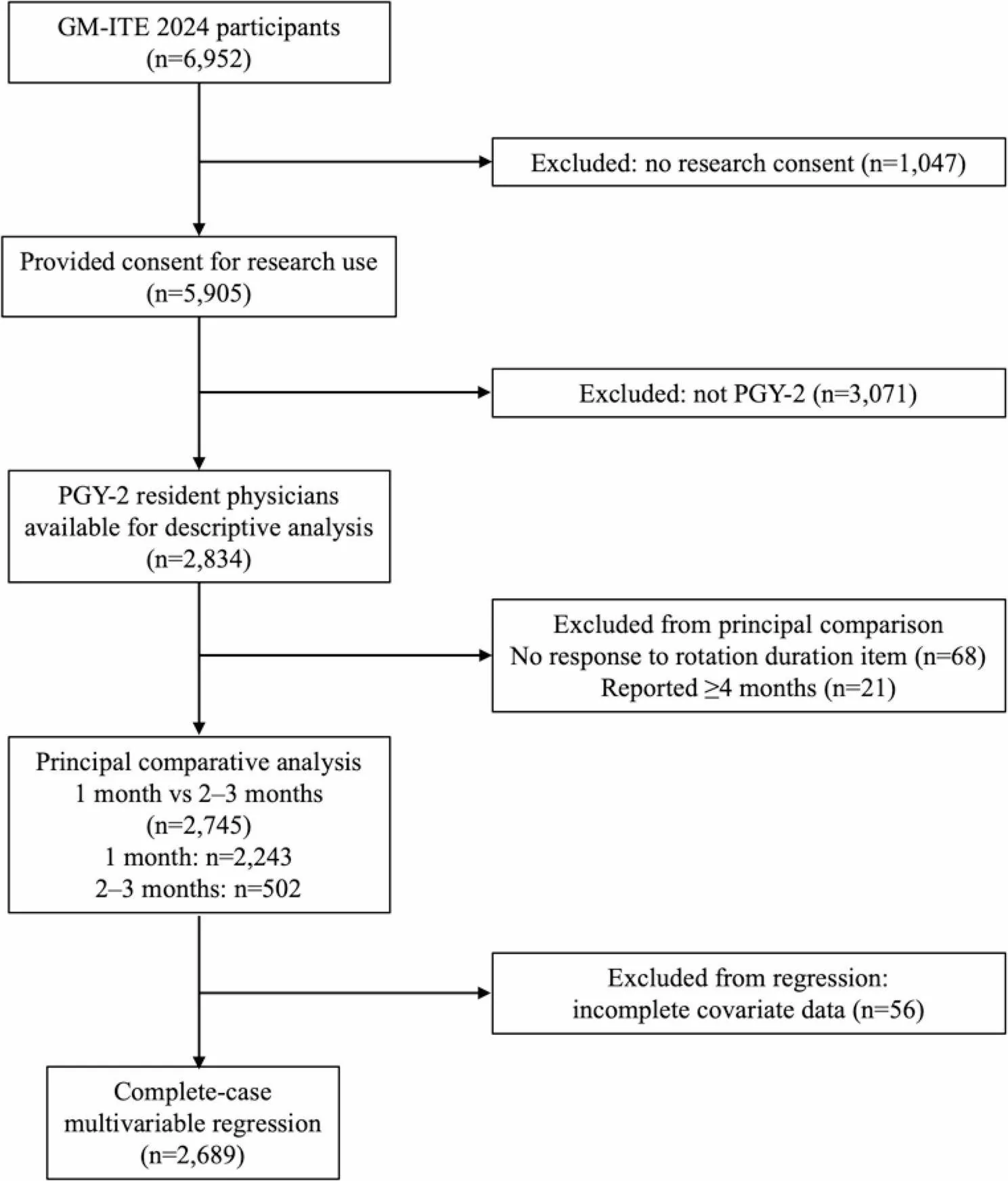
Study flow

This study included 2,834 PGY-2 resident physicians (Fig. 1). Community medicine rotation duration in the overall cohort was one month for 2,243 resident physicians (79.1%), two months for 435 (15.3%), three months for 67 (2.4%), four months for 12 (0.4%), five months for four (0.1%), and six months or more for five (0.2%); data were missing for 68 (2.4%) (Table 1). After restricting the analysis to resident physicians with three months or less of community medicine training, 2,745 resident physicians remained. Among this cohort, 2,243 resident physicians (81.7%) had one month of community medicine training, and 502 (18.3%) had 2–3 months.

**Table 1:** Distribution of community medicine rotation duration among PGY-2 resident physicians (*N* = 2,834)

Duration of community medicine rotation	*n* (%)
1 month	2,243 (79.1)
2 months	435 (15.3)
3 months	67 (2.4)
4 months	12 (0.4)
5 months	4 (0.1)
6 months or more	5 (0.2)
Missing	68 (2.4)

No statistically significant difference in the distribution of one month versus 2–3 months was observed by gender (p = 0.723) (Table 2). By contrast, longer community medicine rotations were more common in university hospitals than in community hospitals (23.3% vs. 17.0%, p = 0.001) (Table 3). When stratified by regional quota status, 2–3-month community medicine rotations were more common among resident physicians with regional quota status than among those without regional quota status (21.8% vs. 17.4%, p = 0.024) (Table 4).

**Table 2:** Gender differences in the distribution of community medicine rotation duration among PGY-2 resident physicians (*N* = 2,745)

Duration of community medicine rotation	Male, *n* (%)	Female, *n* (%)	*p*-value
1 month	1,453 (81.5)	790 (82.1)	0.723
2–3 months	330 (18.5)	172 (17.9)	

Note: P-values were calculated using chi-square tests

**Table 3:** Training program differences in the distribution of community medicine rotation duration among PGY-2 resident physicians (*N* = 2,745)

Duration of community medicine rotation	Community hospital, *n* (%)	University hospital, *n* (%)	*p*-value
1 month	1,818 (83.0)	425 (76.7)	0.001
2–3 months	373 (17.0)	129 (23.3)	

**Table 4:** Community medicine rotation duration by regional quota status among PGY-2 residents with ≤ 3 months’ training (*N* = 2,722)

Duration of community medicine rotation	Non-regional quota status, *n* (%)	Regional quota status, *n* (%)	*p*-value
1 month	1,836 (82.6)	390 (78.2)	0.024
2–3 months	387 (17.4)	109 (21.8)	

### GM-ITE scores according to the community medicine rotation duration

The total GM-ITE score rate was similar between resident physicians with one month and those with 2–3 months of community medicine training (58.36 ± 10.11 vs. 58.50 ± 10.23; p = 0.785; Cohen’s d = 0.01) (Table 5). When the 1-, 2-, and 3-month groups were presented separately, total score rates remained similar across the three groups (58.36 ± 10.11, 58.41 ± 10.35, and 59.09 ± 9.53, respectively; overall p = 0.828) (Table 5). Among the domain-specific correct rates, the general medicine correct rate was nominally higher in the 2–3-month group than in the one-month group (54.43 ± 18.31 vs. 52.49 ± 17.37; p = 0.031; Cohen’s d = 0.11), whereas clinical reasoning, physical examination/procedures, and specific disease correct rates were similar (Table 6. Specialty-specific correct rates did not differ significantly across groups (Table 6). After Benjamini-Hochberg false discovery rate adjustment, the general medicine result was no longer statistically significant (q = 0.122), and no domain- or specialty-specific comparison remained statistically significant (Supplementary file 2).

**Table 5:** GM-ITE total score rate and domain- and specialty-specific correct rates according to community medicine rotation duration. GM-ITE total score rate and domain-specific correct rates

GM-ITE score	1 month (*n* = 2,243) mean ± SD	2 months (*n* = 435) mean ± SD	3 months (*n* = 67) mean ± SD	Overall *p* value (3 groups)	2 m vs. 1 m mean difference (95% CI)	2 m vs. 1 m *p* value	2 m vs. 1 m Cohen’s d	3 m vs. 1 m mean difference (95% CI)	3 m vs. 1 m *p* value	3 m vs. 1 m Cohen’s d
Total score rate	58.36 ± 10.11	58.41 ± 10.35	59.09 ± 9.53	0.828	0.05 (-1.01 to 1.11)	0.931	0.00	0.73 (-1.63 to 3.09)	0.541	0.07
General medicine	52.49 ± 17.37	54.48 ± 18.57	54.10 ± 16.63	0.100	1.99 (0.10 to 3.88)	0.039	0.11	1.61 (-2.50 to 5.73)	0.437	0.09
Clinical reasoning	70.06 ± 12.93	69.53 ± 12.87	72.64 ± 10.78	0.106	-0.53 (-1.85 to 0.79)	0.432	-0.04	2.58 (-0.10 to 5.26)	0.059	0.20
Physical examination / procedures	56.44 ± 14.62	56.14 ± 15.14	55.56 ± 14.51	0.835	-0.30 (-1.85 to 1.25)	0.705	-0.02	-0.89 (-4.47 to 2.70)	0.624	-0.06
Specific disease	54.77 ± 12.09	54.85 ± 11.88	55.18 ± 11.07	0.952	0.08 (-1.15 to 1.30)	0.903	0.01	0.41 (-2.33 to 3.16)	0.765	0.03

*Footnote:* Values are means ± standard deviations unless otherwise stated. Overall p-values were calculated using Welch one-way ANOVA. Pairwise comparisons used Welch t-tests with 1 month as the reference group. Cohen's d values compare each longer-duration group with the 1-month group. Clinical reasoning, physical examination/procedures, and specific disease rows were available in the analytic dataset and are included here as domain-specific outcomes

**Table 6:** GM-ITE total score rate and domain- and specialty-specific correct rates according to community medicine rotation duration. GM-ITE total score rate and specialty-specific correct rates

GM-ITE score	1 month (*n* = 2,243) mean ± SD	2 months (*n* = 435) mean ± SD	3 months (*n* = 67) mean ± SD	Overall *p* value (3 groups)	2 m vs. 1 m mean difference (95% CI)	2 m vs. 1 m *p* value	2 m vs. 1 m Cohen’s d	3 m vs. 1 m mean difference (95% CI)	3 m vs. 1 m *p* value	3 m vs. 1 m Cohen’s d
Total score rate	58.36 ± 10.11	58.41 ± 10.35	59.09 ± 9.53	0.828	0.05 (-1.01 to 1.11)	0.931	0.00	0.73 (-1.63 to 3.09)	0.541	0.07
General medicine	52.49 ± 17.37	54.48 ± 18.57	54.10 ± 16.63	0.100	1.99 (0.10 to 3.88)	0.039	0.11	1.61 (-2.50 to 5.73)	0.437	0.09
Internal medicine	56.88 ± 12.72	56.70 ± 12.80	58.10 ± 12.19	0.686	-0.19 (-1.50 to 1.13)	0.779	-0.01	1.21 (-1.81 to 4.23)	0.425	0.10
Surgery	62.12 ± 17.80	61.55 ± 17.29	60.63 ± 16.32	0.655	-0.56 (-2.35 to 1.22)	0.536	-0.03	-1.48 (-5.53 to 2.56)	0.468	-0.08
Pediatrics	68.89 ± 23.03	68.62 ± 23.54	74.25 ± 19.93	0.094	-0.27 (-2.68 to 2.14)	0.825	-0.01	5.36 (0.41 to 10.31)	0.034	0.23
Obstetrics and gynecology	61.85 ± 21.30	62.30 ± 20.95	63.06 ± 20.58	0.833	0.45 (-1.71 to 2.61)	0.682	0.02	1.21 (-3.88 to 6.30)	0.637	0.06
Psychiatry	63.61 ± 24.62	65.06 ± 24.19	63.81 ± 25.11	0.525	1.45 (-1.05 to 3.94)	0.255	0.06	0.20 (-6.01 to 6.40)	0.950	0.01
Emergency medicine	58.26 ± 14.80	57.70 ± 15.68	56.72 ± 14.88	0.582	-0.55 (-2.15 to 1.04)	0.496	-0.04	-1.54 (-5.22 to 2.14)	0.407	-0.10

*Footnote:* Values are means ± standard deviations unless otherwise stated. Overall *p*-values were calculated using Welch one-way ANOVA. Pairwise comparisons used Welch t-tests with 1 month as the reference group. Cohen's d values compare each longer-duration group with the 1-month group

### Multivariable analysis of the total GM-ITE score rate

In the complete-case multivariable linear regression analysis restricted to resident physicians with 1–3 months of community medicine rotation (n = 2,689), a two-month community medicine rotation was not significantly associated with the total GM-ITE score rate compared with a one-month rotation (adjusted β: 0.44; 95% CI: −0.54 to 1.42; p = 0.382) (Table 7). A small adjusted association was observed for a three-month rotation (adjusted β: 2.33; 95% CI: 0.29 to 4.37; p = 0.025). Because only 65 residents with a three-month rotation were included in the complete-case regression analysis, this estimate should be interpreted cautiously. In sensitivity analyses using hospital-level cluster-robust standard errors, the direction and magnitude of estimates were similar: the three-month estimate for total score rate remained positive (adjusted β: 2.33; 95% CI: 0.61 to 4.06; p = 0.008), whereas the two-month estimate was not statistically significant (adjusted β: 0.44; 95% CI: −0.92 to 1.79; p = 0.527) (Supplementary file 3).

**Table 7:** Factors associated with GM-ITE total score rate in residents with 1–3-month community medicine rotations

Variable	Category	*N*	Mean ± SD	Adjusted β	95% CI	*P*-value
Gender						
	Male	1748	58.72 ± 10.53	(Reference)		
	Female	941	57.79 ± 9.31	-0.95	-1.677 – -0.214	0.011
Age						
	24–26	1155	60.95 ± 9.98	(Reference)		
	27	758	58.37 ± 9.34	-2.24	-3.082 – -1.407	< 0.001
	28+	776	54.60 ± 9.89	-5.91	-6.785 – -5.044	< 0.001
Weekly work hours						
	≤ 60	2178	57.87 ± 9.97	(Reference)		
	60–65	271	61.30 ± 10.27	2.27	1.058–3.474	< 0.001
	65+	240	59.86 ± 10.70	1.30	-0.062–2.668	0.061
On-call / month						
	Zero	49	53.06 ± 11.64	(Reference)		
	1–2 times	548	55.92 ± 9.94	1.78	-1.337–4.899	0.263
	3–5 times	1842	59.07 ± 9.92	2.89	-0.196–5.982	0.066
	More than 6 times	232	60.19 ± 10.61	3.23	-0.096–6.561	0.057
	Unknown	18	55.21 ± 9.08	1.45	-3.727–6.635	0.582
Daily study time						
	None	66	55.68 ± 10.09	(Reference)		
	0–30 min	760	55.52 ± 10.18	-0.75	-3.142–1.646	0.540
	31–60 min	1100	59.01 ± 9.70	2.30	-0.060–4.658	0.056
	61–90 min	513	59.90 ± 9.79	3.07	0.637–5.507	0.013
	91–120 min	157	62.86 ± 9.46	5.47	2.787–8.154	< 0.001
	More than 121 min	93	60.62 ± 11.43	3.98	0.864–7.088	0.012
Community medicine months						
	1 month (1–4 weeks)	2201	58.39 ± 10.11	(Reference)		
	2 months (5–8 weeks)	423	58.32 ± 10.33	0.44	-0.542–1.415	0.382
	3 months (9–12 weeks)	65	59.00 ± 9.52	2.33	0.292–4.371	0.025
Hospital type						
	Community hospital	2143	59.71 ± 9.79	(Reference)		
	University hospital	546	53.23 ± 9.78	-5.12	-6.042 – -4.198	< 0.001
Regional quota status						
	No (Ref)	2194	58.21 ± 10.24	(Reference)		
	Yes	495	59.19 ± 9.58	0.50	-0.408–1.415	0.279

*Footnote:* This complete-case multivariable linear regression analysis was restricted to PGY-2 resident physicians with 1–3 months of community medicine rotation; those with missing rotation duration or ≥4 months of rotation were excluded. N and mean ± SD are unadjusted descriptive statistics. Adjusted coefficients are β coefficients for the GM-ITE total score rate, adjusted for all variables listed in the table, including gender. Robust standard errors were used for 95% confidence intervals and p-values. Reference categories are indicated in the table

Complete-case regression included 2,689 residents, while 56 were excluded due to incomplete covariate data. The total GM-ITE score rate and general medicine correct rate were similar between included and excluded residents, although covariate missingness indicated the possibility of selection bias (Supplementary file 4). In sensitivity analyses restricted to non-university hospitals, the three-month estimate for total score rate remained positive but was not statistically significant (adjusted β: 2.06; 95% CI: −0.34 to 4.47; *p* = 0.093), and the general medicine correct rate showed a small positive association for 2–3 months (adjusted β: 2.82; 95% CI: 0.66 to 4.98; *p* = 0.011) (Supplementary file 5).

## Discussion

This nationwide cross-sectional study examined the distribution of community medicine rotation duration among PGY-2 resident physicians in Japan and its association with GM-ITE performance. Three main findings emerged. First, one month was by far the most common duration of community medicine training, whereas 2–3-month rotations were less common. Second, longer community medicine rotations were more frequently observed in university hospitals and among resident physicians with regional quota status. Third, the unadjusted total GM-ITE score rate was similar between residents with one month and those with 2–3 months of community medicine training; however, in the adjusted analysis that separated two- and three-month rotations, a small adjusted association was observed only for the three-month group, whereas no significant association was observed for the two-month group. In the unadjusted domain-specific analyses, the general medicine correct rate was slightly higher among residents with 2–3 months of community medicine training, but this difference was small and did not remain statistically significant after false discovery rate adjustment; other specialty-specific correct rates were similar between groups. Additional sensitivity analyses using hospital-level cluster-robust standard errors and analyses restricted to non-university hospitals did not materially change this cautious interpretation, although the three-month estimate was less precise in the non-university-only analysis.

These findings should be interpreted in the context of previous GM-ITE research. Prior nationwide studies have shown that general medicine rotations are associated with higher GM-ITE scores, that longer internal medicine rotations are associated with better performance, and that outpatient training may have a nonlinear relationship with GM-ITE performance, with approximately 1 month appearing most advantageous [21, 22, 25]. Taken together, this body of work suggests that associations between training duration and educational outcomes are not uniform across rotation types or outcomes. Our results extend this literature by showing a nuanced pattern in community medicine training: the unadjusted total GM-ITE score rate was similar between residents with 1 month and those with 2–3 months of training, whereas the adjusted analysis, separating two- and three-month rotations, showed a small adjusted association only for the three-month group. Because the three-month group was small, this finding should be interpreted with caution and confirmed in future studies.

International literature on rural and community-oriented training also suggests that the amount and continuity of rural or community exposure may influence perceived preparedness, geographic practice intentions, and subsequent rural practice, although the optimal duration appears to depend on the intended educational or workforce outcome [1–3]. These findings support the view that duration alone should not be assumed to improve all outcomes equally, and that the relevant outcome measure must align with the educational purpose of the rotation.

One possible explanation is that community medicine training offers learning opportunities that are not fully captured by an overall written knowledge examination. Community medicine rotations may emphasize continuity of care, home care, discharge planning, multiprofessional collaboration, and understanding of local care systems [8–18]. These experiences are closely related to community-based medical education, Japan’s community-based integrated care system, and interprofessional education, and may align more closely with broad generalist perspectives than with organ- or specialty-specific knowledge. The observation that the unadjusted difference appeared in the general medicine category, but not in other specialty-specific correct rates, is consistent with this interpretation. At the same time, because this finding was based on an unadjusted comparison, the effect size was small, and the association did not remain statistically significant after false discovery rate adjustment, it should be interpreted as exploratory and not as evidence of educational superiority.

The institutional difference in rotation duration is also noteworthy. University hospitals were more likely than community hospitals to provide 2–3-month community medicine rotations. The introduction of mandatory postgraduate clinical training in Japan was accompanied by changes in residency hospital selection, including a shift of residents from university to non-university hospitals [6]. Previous GM-ITE studies have also reported differences in examination performance between university and community hospitals [24]. Our findings suggest that institutional context may influence not only examination performance but also how programs allocate time to community medicine training. Such differences may reflect variations in program design, affiliation networks, regional placement capacity, or operational constraints rather than educational philosophy alone.

These institutional differences also raise the possibility of program-level confounding and selection bias. Programs offering longer community medicine rotations may systematically differ from those offering shorter rotations in educational culture, resident selection, regional partnerships, clinical placement capacity, supervision systems, and workforce-related goals. Although we adjusted for available variables such as hospital type and regional quota status, unmeasured institutional characteristics may have influenced both rotation duration and GM-ITE performance. To examine this issue, we added hospital-level cluster-robust sensitivity analyses and a non-university-hospital restricted analysis. The cluster-robust analysis yielded similar estimates for the three-month total-score model, whereas the non-university-only analysis showed a positive but non-significant estimate for the three-month group. These findings reduce but do not eliminate concerns about institutional confounding, because unmeasured program-level characteristics may still have influenced both rotation duration and GM-ITE performance.

From an educational implementation perspective, our findings should not be interpreted as evidence that longer community medicine rotations improve overall GM-ITE performance or as direct support for the 8-week recommendation. The Japanese clinical training guideline requires at least 4 weeks of community medicine training and states that 8 weeks or more is desirable [29]. In this policy context, the similar unadjusted total GM-ITE score rates between the one-month and 2–3-month groups provide descriptive information relevant to program planning, but they do not establish equivalence, non-inferiority, or the optimal duration of community medicine training. Because this study was cross-sectional and did not directly assess clinical performance, attitudes, or community-oriented competencies, policy-related implications should remain cautious.

The study also has some limitations. First, the cross-sectional design precludes causal inference. Moreover, residual confounding by resident preference, educational culture, or other unmeasured factors is possible. Second, exposure was based solely on the duration of community medicine training and did not capture the content or quality of training, such as home visits, interprofessional collaboration, supervision, case mix, rural or urban placement, characteristics of host institutions, academic curriculum, or formal classes. Therefore, the duration of the community medicine rotation should be regarded as a crude measure of exposure rather than a standardized, reproducible educational intervention. In addition, because rotation duration was self-reported and categorized, the exact number of weeks and direct compliance with the 8-week recommendation could not be verified. Third, the GM-ITE primarily assesses knowledge and reasoning; it does not directly measure several competencies central to community medicine, such as teamwork, use of community resources, interprofessional collaboration, home care practice, and discharge coordination. This limitation is consistent with established frameworks of assessment in medical education, which distinguish knowledge-based assessment from the assessment of clinical skills, competence, and performance in authentic practice settings [30, 31]. Fourth, although we conducted hospital-level cluster-robust and non-university-hospital sensitivity analyses, unmeasured institutional characteristics and program-level confounding may remain. Fifth, complete-case analysis excluded 56 residents with incomplete covariate data; score outcomes were similar between included and excluded residents, but selection bias due to covariate missingness cannot be ruled out. Sixth, the analysis was based on a single examination year and a PGY-2 cohort; therefore, reproducibility across years should be confirmed in future studies. Finally, the number of residents with a three-month rotation was small, and the number with more than three months was very small, limiting our ability to examine longer rotations robustly.

## Conclusions

In this nationwide cross-sectional study of PGY-2 resident physicians in Japan, a 1-month duration was the predominant length of community medicine rotation, and longer rotations were more common in university hospitals and among residents with regional quota status. In unadjusted comparisons, the total GM-ITE score rate was similar between the one-month and 2–3-month groups. In adjusted analyses, a small association was observed for the three-month group but not for the two-month group; however, this finding should be interpreted with caution, given the small subgroup size, cross-sectional design, and potential residual and institutional confounding. Residents with 2–3 months of training showed slightly higher unadjusted performance in the general medicine category; however, the effect size was small, and this result did not remain statistically significant after false discovery rate adjustment. The finding should be considered exploratory. These results contribute to discussions about the duration of community medicine rotations but do not establish an optimal duration or demonstrate improvements in broader community medicine competencies. Future research should examine the content, quality, and timing of community medicine training and evaluate broader educational outcomes beyond written examination scores.

## Supplementary Information

Supplementary Material 1. Supplementary file 1. The GM-ITE 2024 background questionnaire items used in this study.

Supplementary Material 2. Supplementary file 2. Benjamini-Hochberg false discovery rate adjustment for exploratory domain- and specialty-specific correct-rate comparisons.

Supplementary Material 3. Supplementary File 3. Multivariable regression analyses using hospital-level cluster-robust standard errors.

Supplementary Material 4. Supplementary file 4. Comparison of residents included in complete-case regression with those excluded because of incomplete covariate data.

Supplementary Material 5. Supplementary file 5. Sensitivity analyses restricted to non-university hospitals.

## Data Availability

No datasets were generated or analysed during the current study.

## Abbreviations

CI: confidence interval
GM-ITE: General Medicine In-Training Examination
JAMEP: Japan Institute for Advancement of Medical Education Program
PGY: postgraduate year
STROBE: Strengthening the Reporting of Observational Studies in Epidemiology
