# Supplementary material for: Association between community medicine rotation duration and general medicine in-training examination scores among second-year resident physicians in Japan: a nationwide cross-sectional study
Source: BMC Med Educ. 2026 Jul 7;26:1546. doi: 10.1186/s12909-026-09897-y (PMC13625247; doi:10.1186/s12909-026-09897-y)
Supplement: Supplementary file 1 [file 12909_2026_9897_MOESM1_ESM.docx]

# **Supplementary file 1.** The GM-ITE 2024 background questionnaire items used in this study.

| **Question / variable** | **Response options or format** |
| --- | --- |
| Consent for research use | Yes / No. |
| Postgraduate year | PGY-1 / PGY-2 / Other. |
| Duration of community medicine rotation during initial residency | 1 month (1–4 weeks); 2 months (5–8 weeks); 3 months (9–12 weeks); 4 months (13–16 weeks); 5 months (17–20 weeks); 6 months or more (≥21 weeks). |
| Age | Age in years. |
| Gender | Male / Female / Do not reply. |
| Weekly work hours | Weekly work hours were originally collected using 11 response categories ranging from “less than 40 hours” to “100 hours or more,” with approximate monthly overtime hours provided for each category. For analysis, these responses were categorized as ≤60 hours, 60–65 hours, and ≥65 hours. |
| On-call frequency per month | Zero; 1–2 times; 3–5 times; More than 6 times; Unknown. |
| Daily self-study time | None; 0–30 minutes; 31–60 minutes; 61–90 minutes; 91–120 minutes; More than 121 minutes. |
| Regional quota status | Yes / No. |

Variables not obtained from the background questionnaire: GM-ITE examination scores were obtained from the examination database. Hospital type was obtained from the GM-ITE administrative database and classified as community hospital or university hospital.

**Note**: The items below are an English translation of the background questionnaire items relevant to the present analysis. The questionnaire was administered as part of the General Medicine In-Training Examination (GM-ITE) 2024 by the Japan Institute for Advancement of Medical Education Program (JAMEP). The original questionnaire was administered in Japanese.
