# Supplementary material for: Association between community medicine rotation duration and general medicine in-training examination scores among second-year resident physicians in Japan: a nationwide cross-sectional study
Source: BMC Med Educ. 2026 Jul 7;26:1546. doi: 10.1186/s12909-026-09897-y (PMC13625247; doi:10.1186/s12909-026-09897-y)
Supplement: Supplementary file 2 [file 12909_2026_9897_MOESM2_ESM.docx]

**Supplementary file 2.** Benjamini-Hochberg false discovery rate adjustment for exploratory domain- and specialty-specific correct-rate comparisons.

**Supplementary Table S2a.** Benjamini-Hochberg false discovery rate adjustment for exploratory domain-specific correct-rate comparisons.

| **Outcome** | **1 month,**  **(mean ± SD)** | **2–3 months,**  **(mean ± SD)** | **Mean difference**  **(95% CI)** | **Unadjusted**  **p value** | **FDR-adjusted**  **q value** | **Nominal p<0.05** | **FDR**  **q<0.05** |
| --- | --- | --- | --- | --- | --- | --- | --- |
| General medicine | 52.49 ± 17.37 | 54.43 ± 18.31 | 1.94 (0.18 to 3.70) | 0.031 | 0.122 | Yes | No |
| Clinical reasoning | 70.06 ± 12.93 | 69.94 ± 12.64 | -0.12 (-1.35 to 1.12) | 0.854 | 0.854 | No | No |
| Physical examination/procedures | 56.44 ± 14.62 | 56.06 ± 15.04 | -0.38 (-1.83 to 1.07) | 0.610 | 0.854 | No | No |
| Specific disease | 54.77 ± 12.09 | 54.89 ± 11.77 | 0.12 (-1.03 to 1.27) | 0.836 | 0.854 | No | No |

*Footnote:* Values are presented as means ± standard deviations. Mean differences and 95% CIs are shown as 2–3 months minus 1 month and were calculated using Welch’s t-tests. Benjamini-Hochberg false discovery rate adjustment was applied to the four domain-specific outcomes. GM-ITE total score rate was treated as the primary outcome and was not included in the FDR correction. FDR, false discovery rate; GM-ITE, General Medicine In-Training Examination; SD, standard deviation.

**Supplementary Table S2b.** Benjamini-Hochberg false discovery rate adjustment for exploratory specialty-specific correct-rate comparisons.

| **Outcome** | **1 month,**  **(mean ± SD)** | **2–3 months,**  **(mean ± SD)** | **Mean difference**  **(95% CI)** | **Unadjusted**  **p value** | **FDR-adjusted**  **q value** | **Nominal p<0.05** | **FDR**  **q<0.05** |
| --- | --- | --- | --- | --- | --- | --- | --- |
| Internal medicine | 56.88 ± 12.72 | 56.88 ± 12.72 | -0.00 (-1.23 to 1.23) | 0.999 | 0.999 | No | No |
| Surgery | 62.12 ± 17.80 | 61.43 ± 17.15 | -0.69 (-2.36 to 0.99) | 0.421 | 0.809 | No | No |
| Pediatrics | 68.89 ± 23.03 | 69.37 ± 23.15 | 0.48 (-1.76 to 2.72) | 0.674 | 0.809 | No | No |
| Obstetrics and gynecology | 61.85 ± 21.30 | 62.40 ± 20.88 | 0.55 (-1.48 to 2.58) | 0.594 | 0.809 | No | No |
| Psychiatry | 63.61 ± 24.62 | 64.89 ± 24.29 | 1.28 (-1.08 to 3.64) | 0.287 | 0.809 | No | No |
| Emergency medicine | 58.26 ± 14.80 | 57.57 ± 15.56 | -0.69 (-2.18 to 0.81) | 0.368 | 0.809 | No | No |

*Footnote:* Values are presented as means ± standard deviations. Mean differences and 95% CIs are shown as 2–3 months minus 1 month and were calculated using Welch’s t-tests. Benjamini-Hochberg false discovery rate adjustment was applied to the six specialty-specific outcomes. FDR, false discovery rate; GM-ITE, General Medicine In-Training Examination; SD, standard deviation.
