# Supplementary material for: Association between community medicine rotation duration and general medicine in-training examination scores among second-year resident physicians in Japan: a nationwide cross-sectional study
Source: BMC Med Educ. 2026 Jul 7;26:1546. doi: 10.1186/s12909-026-09897-y (PMC13625247; doi:10.1186/s12909-026-09897-y)
Supplement: Supplementary file 3 [file 12909_2026_9897_MOESM3_ESM.docx]

**Supplementary File 3.** Multivariable regression analyses using hospital-level cluster-robust standard errors.

| **Outcome** | **Model** | **Reference** | **Comparison** | **N** | **No. hospitals/clusters** | **Adjusted β** | **Cluster-robust SE** | **95% CI lower** | **95% CI upper** | **p value** |
| --- | --- | --- | --- | --- | --- | --- | --- | --- | --- | --- |
| GM-ITE total score rate | Model A:  1 vs 2–3 months | 1 month | 2–3 months | 2689 | 544 | 0.68 | 0.63 | -0.55 | 1.91 | 0.280 |
| General medicine correct rate | Model A:  1 vs 2–3 months | 1 month | 2–3 months | 2689 | 544 | 2.68 | 0.97 | 0.76 | 4.59 | 0.006 |
| GM-ITE total score rate | Model B:  1/2/3 months | 1 month | 2 months | 2689 | 544 | 0.44 | 0.69 | -0.92 | 1.79 | 0.527 |
| GM-ITE total score rate | Model B:  1/2/3 months | 1 month | 3 months | 2689 | 544 | 2.33 | 0.88 | 0.61 | 4.06 | 0.008 |
| General medicine correct rate | Model B:  1/2/3 months | 1 month | 2 months | 2689 | 544 | 2.57 | 1.07 | 0.48 | 4.67 | 0.016 |
| General medicine correct rate | Model B:  1/2/3 months | 1 month | 3 months | 2689 | 544 | 3.37 | 2.14 | -0.84 | 7.58 | 0.117 |

*Footnote:* Models were adjusted for age, gender, weekly work hours, on-call frequency, self-study time, hospital type, regional quota status, and community medicine rotation duration. The analysis population comprised residents with 1–3 months of community medicine rotation and complete covariate data. Hospital-level cluster-robust standard errors were calculated using the hospital identifier as the clustering unit.
