# Supplementary material for: Association between community medicine rotation duration and general medicine in-training examination scores among second-year resident physicians in Japan: a nationwide cross-sectional study
Source: BMC Med Educ. 2026 Jul 7;26:1546. doi: 10.1186/s12909-026-09897-y (PMC13625247; doi:10.1186/s12909-026-09897-y)
Supplement: Supplementary file 4 [file 12909_2026_9897_MOESM4_ESM.docx]

**Supplementary file 4.** Comparison of residents included in complete-case regression with those excluded because of incomplete covariate data.

| **Variable** | **Category/**  **scale** | **Complete-case regression (n=2,689)** | **Excluded due to incomplete covariates (n=56)** | **p value** | **SMD** | **Missing data source** |
| --- | --- | --- | --- | --- | --- | --- |
| Age | 24–26 years | 1,155 (43.0%) | 17 (30.4%) | <0.001 | 0.933 | Age,  missing: 20 |
|  | 27 years | 758 (28.2%) | 11 (19.6%) | <0.001 | 0.933 |  |
|  | 28+ years | 776 (28.9%) | 8 (14.3%) | <0.001 | 0.933 |  |
| Gender | Male | 1,748 (65.0%) | 35 (62.5%) | 0.697 | 0.052 | None |
|  | Female | 941 (35.0%) | 21 (37.5%) | 0.697 | 0.052 |  |
| Weekly work hours | ≤60 | 2,178 (81.0%) | 40 (71.4%) | <0.001 | 0.516 | Weekly work hours,  missing: 7 |
|  | 60–65 | 271 (10.1%) | 3 (5.4%) | <0.001 | 0.516 |  |
|  | 65+ | 240 (8.9%) | 6 (10.7%) | <0.001 | 0.516 |  |
| On-call / month | Zero | 49 (1.8%) | 1 (1.8%) | <0.001 | 0.332 | On call month,  missing: 3 |
|  | 1–2 times | 548 (20.4%) | 7 (12.5%) | <0.001 | 0.332 |  |
|  | 3–5 times | 1,842 (68.5%) | 41 (73.2%) | <0.001 | 0.332 |  |
|  | More than 6 times | 232 (8.6%) | 4 (7.1%) | <0.001 | 0.332 |  |
|  | Unknown | 18 (0.7%) | 0 (0.0%) | <0.001 | 0.332 |  |
| Daily study time | None | 66 (2.5%) | 1 (1.8%) | <0.001 | 0.476 | Study time,  missing: 6 |
|  | 0–30 min | 760 (28.3%) | 16 (28.6%) | <0.001 | 0.476 |  |
|  | 31–60 min | 1,100 (40.9%) | 23 (41.1%) | <0.001 | 0.476 |  |
|  | 61–90 min | 513 (19.1%) | 4 (7.1%) | <0.001 | 0.476 |  |
|  | 91–120 min | 157 (5.8%) | 2 (3.6%) | <0.001 | 0.476 |  |
|  | ≥121 min | 93 (3.5%) | 4 (7.1%) | <0.001 | 0.476 |  |
| Community medicine rotation | 1 month | 2,201 (81.9%) | 42 (75.0%) | 0.439 | 0.167 | None |
|  | 2 months | 423 (15.7%) | 12 (21.4%) | 0.439 | 0.167 |  |
|  | 3 months | 65 (2.4%) | 2 (3.6%) | 0.439 | 0.167 |  |
| GM-ITE total score rate | mean ± SD | 58.39 ± 10.12 | 57.97 ± 10.45 | 0.767 | 0.041 | None |
| General medicine correct rate | mean ± SD | 52.85 ± 17.54 | 52.46 ± 18.83 | 0.878 | 0.022 | None |

*Footnote:* Categorical variables are n (%), and continuous variables are mean ± SD. P values compare included versus excluded residents and are not level-specific tests. SMD for categorical variables is the maximum absolute level-specific standardized mean difference. Categorical variables were compared using chi-square tests; Fisher exact or simulated chi-square tests were used when expected counts were small. Continuous variables were compared using Welch’s t-tests. Excluded residents accounted for 56/2,745 (2.0%). Larger SMDs mainly reflect missingness in covariates that defined exclusion. Complete-case n=2,689; excluded because of missing model variables n=56.
