# Supplementary material for: Association between community medicine rotation duration and general medicine in-training examination scores among second-year resident physicians in Japan: a nationwide cross-sectional study
Source: BMC Med Educ. 2026 Jul 7;26:1546. doi: 10.1186/s12909-026-09897-y (PMC13625247; doi:10.1186/s12909-026-09897-y)
Supplement: Supplementary file 5 [file 12909_2026_9897_MOESM5_ESM.docx]

**Supplementary file 5.** Sensitivity analyses restricted to non-university hospitals.

| **Outcome** | **Model** | **Reference** | **Comparison** | **N** | **No. hospitals/clusters** | **Adjusted β** | **SE** | **95% CI lower** | **95% CI upper** | **p value** |
| --- | --- | --- | --- | --- | --- | --- | --- | --- | --- | --- |
| GM-ITE total score rate | Model A:  1 vs 2–3 months | 1 month | 2–3 months | 2,143 | 494 | 0.73 | 0.75 | -0.74 | 2.20 | 0.331 |
| General medicine correct rate | Model A:  1 vs 2–3 months | 1 month | 2–3 months | 2,143 | 494 | 2.82 | 1.1 | 0.66 | 4.98 | 0.011 |
| GM-ITE total score rate | Model B:  1/2/3 months | 1 month | 2 months | 2,143 | 494 | 0.59 | 0.81 | -1.01 | 2.18 | 0.469 |
| GM-ITE total score rate | Model B:  1/2/3 months | 1 month | 3 months | 2,143 | 494 | 2.06 | 1.22 | -0.34 | 4.47 | 0.093 |
| General medicine correct rate | Model B:  1/2/3 months | 1 month | 2 months | 2,143 | 494 | 2.94 | 1.18 | 0.63 | 5.25 | 0.013 |
| General medicine correct rate | Model B:  1/2/3 months | 1 month | 3 months | 2,143 | 494 | 1.70 | 2.68 | -3.56 | 6.96 | 0.525 |

*Footnote:* The analysis was restricted to residents from non-university hospitals with 1–3 months of community medicine rotation and complete covariate data. Models were adjusted for the same covariates as the main model, except hospital type, which was omitted because the analysis population was restricted to non-university hospitals. Hospital-level cluster-robust standard errors were calculated using Hospital as the clustering unit. The three-month subgroup was small; therefore, estimates for this subgroup should be interpreted cautiously.
